# Integrative Phosphoproteomic and Proteomic Analysis of Exposed to Oxidative Stress

**DOI:** 10.1021/acs.jproteome.5c00137

**Published:** 2025-06-02

**Authors:** Víctor Arribas, Ana Borrajo, María Luisa Hernáez, Raquel Martínez, Lucía Monteoliva, Concha Gil, Gloria Molero

**Affiliations:** † Department of Microbiology and Parasitology, Faculty of Pharmacy, Complutense University of Madrid (UCM), 28040 Madrid, Spain; ‡ Proteomics Unit, Biological Techniques Center, Complutense University of Madrid (UCM), 28040 Madrid, Spain

**Keywords:** C. albicans, phosphoproteomics, proteomics, oxidative stress response, protein kinases, transcription factors, Cdc5, Kis1, Gzf3

## Abstract

is an opportunistic pathogen, which has recently been included in the high-priority list of pathogenic fungi by the World Health Organization (WHO). The scarce arsenal available to treat such invasive fungal infections makes the discovery of new antifungal targets an important task. This study utilizes DDA-MS technology to investigate both the phosphoproteomics and proteomics of during its late-stage response to oxidative stress induced by H_2_O_2_, aiming to identify key proteins involved. Phosphorylation, as an important post-translational modification, plays a crucial role in the ability of to survive oxidative stress. Our study enabled the identification and quantification of important changes in both protein abundance and phosphorylation events across multiple proteins following a 200 min 10 mM H_2_O_2_ treatment. The use of the DDA-MS approach allowed for the identification of new actors in the response to oxidative stress. Novel phosphorylation sites were identified in kinases and transcription factors. Regarding protein kinases, Cdc5-reduced phosphorylation may mediate a transient G2 cell cycle arrest, while Kis1—the regulatory β-subunit of Snf1 kinase—might play a role in ROS scavenging following oxidative stress. In terms of transcription factors, Gzf3-decreased phosphorylation was essential for cell survival and ROS detoxification after oxidative stress.

## Graphical abstract

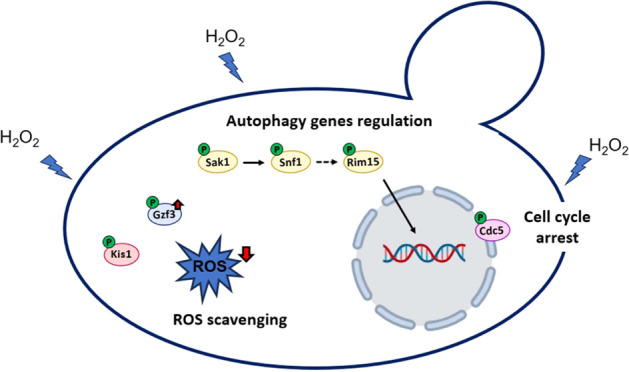

## Introduction

 is a common fungus of the human microbiota that, under specific circumstances, can turn into a pathogen—particularly in individuals with a weakened immune system, leading, under certain circumstances, to invasive candidiasis (IC). IC can also arise in patients undergoing abdominal surgery or using medical devices.^1,2^ The incidence of IC has been increasing annually, and for this reason, the World Health Organization (WHO) has included as a priority in the fungal pathogen list.^3,4^

During infection, macrophages interact with and try to phagocytize it, initiating a respiratory burst which produces reactive oxygen species (ROS) such as hydrogen peroxide (H_2_O_2_).^5–7^ The survival of depends on its ability to swiftly respond to oxidative stress by promoting changes in the abundances of proteins and modulating different mechanisms such as proteasome activity.^8^ Proteome remodulation involves changes in the abundance of key proteins of the three main ROS scavenging systems, as well as other proteins (e.g., Prn1 or Cub1) which are essential for cell survival following oxidative stress.^9^

 finely tunes the activation of diverse signaling pathways through several phosphorylation events. Protein phosphorylation is crucial for signaling by changing their conformation, stability, activity, subcellular location, or interactions with other proteins. Phosphoproteomics is a very useful tool for the discovery of the phosphosites involved in these processes.^10,11^ The combination of proteomics and phosphoproteomics allows for an integrative study of the changes induced by a certain stimulus.

In , the high osmolarity glycerol (HOG) MAPK pathway stands out as the primary signaling pathway for sensing oxidative stress. Exogenous H_2_O_2_ promotes Ssk1 phosphorylation—a key regulator in the pathway, which, in turn, activates the MAPKKK Ssk2 via phosphorylation. This process triggers a kinase cascade that ultimately leads to activation of the Hog1MAPK.^12,13^ Hog1 phosphorylation also promotes activation of the cell wall integrity MAPK Mkc1, although this event is not critical for oxidative stress signaling.^14^ Transcription factors also play a key role in the oxidative stress response, with Cap1 being the main regulator in .^15^ Cap1 undergoes phosphorylation within the nucleus, which activates the expression of different antioxidant genes.^16^ Upon activation, Cap1 also regulates the expression of other transcription factors, such as Gzf3 and Fcr1.^17,18^

Although MAPK signaling after oxidative stress occurs within the first minutes, at 200 min, has not yet fully recovered. It continues to suffer from elevated ROS levels and responds with various detoxification mechanisms.^8^ This late response also involves multiple phosphorylation events that regulate cell recovery, which remain poorly understood in . TORC1 kinase participates in this response by regulating autophagy through Snf1 kinase.^19^ While the Snf1 catalytic subunit and the regulatory β-subunit Kis1 ^20^ presented phosphorylation sites by TORC1 in ,^21–23^ no phosphosites have been identified in . Synchronized cells also modulate the cell cycle progression in response to oxidative stress,^24^ with Cdc5 kinase playing a pivotal role in cell cycle progression—particularly in spindle assembly and mitotic exit.^25^ In , this kinase is subject to different post-translational regulations by Cdk1 in response to osmotic stress or DNA damage, in order to regulate the cell cycle progression.^26–28^ Consequently, phosphorylation stands out as a very important mechanism to control the response of to oxidative stress.

In our previous work on the changes in protein abundance in after a 200 min 10 mM H_2_O_2_ treatment using DIA (Data-independent acquisition)-MS technology,^8^ we unveiled changes in antioxidative mechanisms, the proteasome, and other cellular processes. In the present work, to unveil phosphorylation events involved in the recovery of in the later oxidative stress response, we conducted an integrative proteomic and phosphoproteomic assay in the SC5314 strain after a 200 min of 10 mM H_2_O_2_ treatment. Our results demonstrate that, after 200 min of the oxidative stress,presents significant changes in the phosphorylation status of many proteins. Some of the identified phosphosites are consistent with those described for , while others have been newly detected. Our integrative analysis suggests that phosphorylation regulates ribosome components and different processes already associated with the oxidative stress response—including proteasome activation and DNA repair—while also implicating new pathways such as oxidative phosphorylation. Notably, our study reveals changes in the phosphorylation status of some kinases and transcription factors after 200 min of exposure to 10 mM H_2_O_2_. These modifications promote the survival of , indicating that they are potential new targets for antifungals. This study is the first integrative proteomic and phosphoproteomic investigation of the oxidative stress response in .

## Experimental Procedures

### Fungal Strains and Culture Conditions

 wild-type SC5314 was used for proteomic and phosphoproteomic assays. wild-type strain (SN250) and the corresponding null mutants in kinase and transcription factor strains were obtained from Noble’s collection.^29^ Cells were grown in YPD-rich medium (1% yeast extract, 2% peptone, and 2% glucose) at 30 °C until reaching the exponential phase (OD_600_ = 0.8–1). Later, 10 mM H_2_O_2_ was added, and cultures were incubated again at 30 °C in rotatory shaking (180 rpm). For growth curves, culture at early exponential phase (OD_600_ = 0.4) was incubated with 10 mM H_2_O_2_ for 12 h at 30 °C with shaking. Regarding the drop growth assay, cells were grown to reach the exponential phase (OD_600_= 0.8–1) and later were cultured in YPD solid medium supplemented with 5 mM H_2_O_2_.

### Experimental Design

 SC5314 was cultured until reaching the exponential phase and then incubated with or without 10 mM H_2_O_2_ for 200 min. A total of 8 samples from 4 biological replicates of each condition (control or treated) were obtained to perform the integrative DDA-MS proteomic and phosphoproteomic assay. Analysis for significant protein abundance changes between treated and their respective control samples was performed by applying statistical nested *t* test applying *p* value <0.05.

### Cell Disruption and Protein Extracts Quantification

Control and treated cells were harvested and washed three times in PBS. Cells were then resuspended in a lysis buffer containing 50 mM Tris-HCl (pH 7.5), 1 mM EDTA, 1 mM DTT, 150 mM NaCl, 10% protease inhibitors (Pierce), and 5 mM phenylmethylsulfonyl fluoride (PMSF). Cells were disrupted by mechanical shaking with glass beads (0.5–0.75 mm diameter), applying 5 cycles of 20 s in a Fast-Prep system (Bio101; Savant). Samples were centrifuged at 13,000 rpm for 15 min to obtain the cell extracts from the supernatant. Finally, protein concentrations were measured using the Bradford assay.

### Proteomic and Phosphoproteomic Assay

Peptide digestion was performed after protein precipitation with methanol/chloroform using 300 μg of protein extracts. In a few words, samples were reduced with 10 mM DTT at 37 °C for 30 min and then alkylated with 55 mM Iodacetamide for 20 min in darkness. Finally, 1/10 of recombinant grade trypsin was added to each sample (Roche Molecular Biochemicals) in 25 mM ammonium bicarbonate (pH 8.5) and incubated overnight at 37 °C. 30 μg (one-tenth) of the sample was retained for further analysis of the peptides. The sample was acidified and dried by vacuum centrifugation (SpeedVac, Savant, Thermo Fisher). It was reconstituted in 12 μL of loading buffer (2% ACN, 0.1% formic acid (FA)), and peptides were quantified by fluorimetry using a Qubit4 system (Thermo Scientific).

The rest of the peptides were added to a freshly prepared sample loading buffer in a ratio 1:10 (v/v) containing 80% ACN, 5% TFA, and 1 M glycolic acid (catalog no. 12473-7, Sigma-Aldrich). The peptides were then incubated with TiO_2_ beads (5 μ Titansphere, GL Sciences, Tokyo, Japan) at a peptides/TiO_2_ ratio of 1:6 (*w/w*) for 15 min at room temperature. The incubated beads were washed with 80% ACN and 1% TFA and then with 20% ACN and 0.1% TFA. The bound peptides were eluted twice using an ammonia solution at pH 11.5. The sample was acidified and dried by vacuum centrifugation (SpeedVac, Savant, Thermo Fisher). It was reconstituted in 12 μL of loading buffer (2% ACN, 0.1% formic Acid (FA)), and peptides were quantified by fluorimetry using a Qubit4 system (Thermo Scientific).

The peptides (1 μg) were analyzed via liquid nanochromatography (nano Easy-nLC 1000, Thermo Scientific, Bremen, Germany) coupled to a Q-Exactive HF high-resolution mass spectrometer (Thermo Scientific). The peptides were concentrated online via reversed-phase chromatography using an Acclaim PepMap 100 guard column (Thermo Scientific) and were then separated on a Picofrit C18 reversed-phase analytical column (Thermo Scientific). MS/MS data were acquired in the data-dependent acquisition (DDA) mode of the MS. Mass spectra were acquired in a Q-Exactive HF hybrid quadrupole-Orbitrap mass spectrometer (Thermo Scientific) in a full-MS data-dependent acquisition (DDA) in a positive mode with Xcalibur 4.5 software. MS scans were acquired at *m*/*z* range of 350–1800 Da followed by data-dependent MS/MS scan (with a threshold of 0.01) of the 15 most abundance precursors with charges of 2–5 in MS scans for high-energy collision dissociation (HCD) fragmentation with a dynamic exclusion of 10 s and normalized collision energy (NCE) of 20. Peptide spectrum matches were filtered to a false-discovery rate (FDR) of >1%. Mass spectra (raw files) were processed using Proteome Discoverer v2.2 software (Thermo Scientific) with the MASCOT v.2.8 search engine, using the *Candida* Genome Database (release 2020_06, 6209 sequences) (CGD)^24^ for protein identification to generate a sample-specific peptide list. Search parameters included carbamidomethylation of cysteines as fixed modification, oxidation of methionine, N-terminal acetylation as variable modifications, and for phosphoproteomics analysis, phosphorylation of serines, treonines, and tyrosines as variable modifications were added, trypsin as enzyme, and a maximum of 1 or 2 missed cleavages allowed for proteomics or phosphoproteomics, respectively. The precursor mass tolerance was 10 ppm, and the fragment mass tolerance was 0.02 Da. The validation was based on q-value from Percolator algorithm, with an FDR > 0.01.

### Peptides Quantification

To determine the abundance of the identified peptides in different isolates, a label-free experiment based on the precursor signal intensity was performed. The processing workflow was initiated with the recalibration of masses through a rapid search in Sequest HT (Thermo Fisher Scientific, Inc.) against the database, and based on the positive identifications, an alignment of the chromatograms of all the samples with a tolerance of up to 10 min. Subsequently, the alignment of the retention times between the different samples analyzed for the quantification of the precursor ions was performed, considering the unique peptides present in at least 50% of the replicates. Finally, the results were normalized to the total amount of the peptides, equaling the total abundance among the different samples.

### Bioinformatic Analysis of Proteins Function, Interaction Network, and Signaling Pathways

GO enrichment analysis was performed using the GO Term Finder and GO Slim Mapper tools from *Candida* Genome Database (CGD) grouped by biological process, molecular function.^30^ KEGG BRITE software was also used for protein categorization. Protein clustering analysis and protein interaction network were performed using STRING v.12.0 software. Protein assignment to their respective signaling pathways was carried out using KEGG Mapper software.

### Viability Assays

 null mutant and wild-type strain cells were harvested after 200 min with 10 mM H_2_O_2_. Later, 5 μL of propidium iodide (PI) (50 μg/mL) was added for 5 min at room temperature and then washed twice with PBS. PI positive death cells were measured by flow cytometry using with FlowJo software. Statistical analysis was performed using a *t* test.

### ROS Detection

Yeast cells were cultured with or without 10 mM H_2_O_2_ for 200 min and then washed three times with PBS. Cells were stained with dihydrorhodamine 123 (DHR123) (Sigma) at 5 mg/mL final concentration for 20 min to detect intracellular ROS and followed by two PBS washes. ROS-positive cells were detected by flow cytometry and analyzed with FlowJo software. Statistical analysis was performed using a *t* test. Cells were also observed by fluorescence microscopy.

### MAPK Phosphorylation

Hog1 and Mkc1 phosphorylation levels were measured by Western blotting in the SC5314 strain treated with 10 mM H_2_O_2_. Samples were collected after 15, 50, 100, and 200 min of treatment. Cell extracts were prepared as previously described, and protein concentrations were measured using the Bradford assay (Protein Assay Dye Reagent Concentrate, Bio-Rad). Samples were separated by SDS-PAGE and transferred to a nitrocellulose membrane (GE Healthcare). Phosphorylated Hog1 (Hog1-P) and Mkc1 (Mkc1-P) were detected using p38 MAPK and p44/42 MAPK antibodies, respectively, followed by incubation with Alexa Fluor Plus 800 and Alexa Fluor Plus 680 secondary antibodies. Signals were detected using Odyssey Fc Imaging System (LI-COR Biosciences) and quantified using ImageJ (v1.54). Statistical analysis was performed on three different replicates using a paired *t* test.

### Cell Cycle Analysis

 SC5314 strain cells were cultured to the exponential phase (OD_600_= 0.8–1) and incubated with or without 10 mM H_2_O_2_. Samples were collected from 0 to 6 h of treatment. Cells were fixed in 70% ethanol, washed twice, and resuspended in PBS. Next, 10 μL of RNase (10 mg/mL) was added for 10 min at 37 °C, and the cells were washed again twice with PBS. Finally, 5 μL of PI (50 μg/mL) was added for 5 min at room temperature, followed by two additional PBS washes. Cell cycle was performed by measuring DNA content using flow cytometry and analyzed with FlowJo software.

### Data Availability

The data set from proteomic analysis has been deposited in the ProteomeXchange Consortium via the PRIDE partner repository^31^ with the data set identifier PXD035231 and 10.6019/PXD035231.

## Results

### Integrative Analysis of the Phosphoproteomic and Proteomic Oxidative Stress Response

We performed an integrative quantitative proteomic and phosphoproteomic assay to simultaneously measure the changes in the abundance of peptides and phosphopeptides in the SC5314 wild-type strain after a 200 min 10 mM H_2_O_2_ treatment (Figure 1A). The proteomic comparison between treated and nontreated conditions using DDA technology allowed for the identification of 3852 proteins, with 3805 being quantified (Table S1). Statistical analyses (*p*-value <0.05 and fold change >2.5) of the relative quantification between both conditions revealed 182 proteins that had significantly increased in abundance and 103 proteins that significantly decreased (Figure 1B and Table S2). The proteins with significant changes in abundance are presented as volcano plots (Figure 1B). In particular, proteins that were significantly increased in abundance in the proteomic assay were significantly enriched in oxidoreductase activity and proteasomal protein catabolic processes.

**Figure 1:**
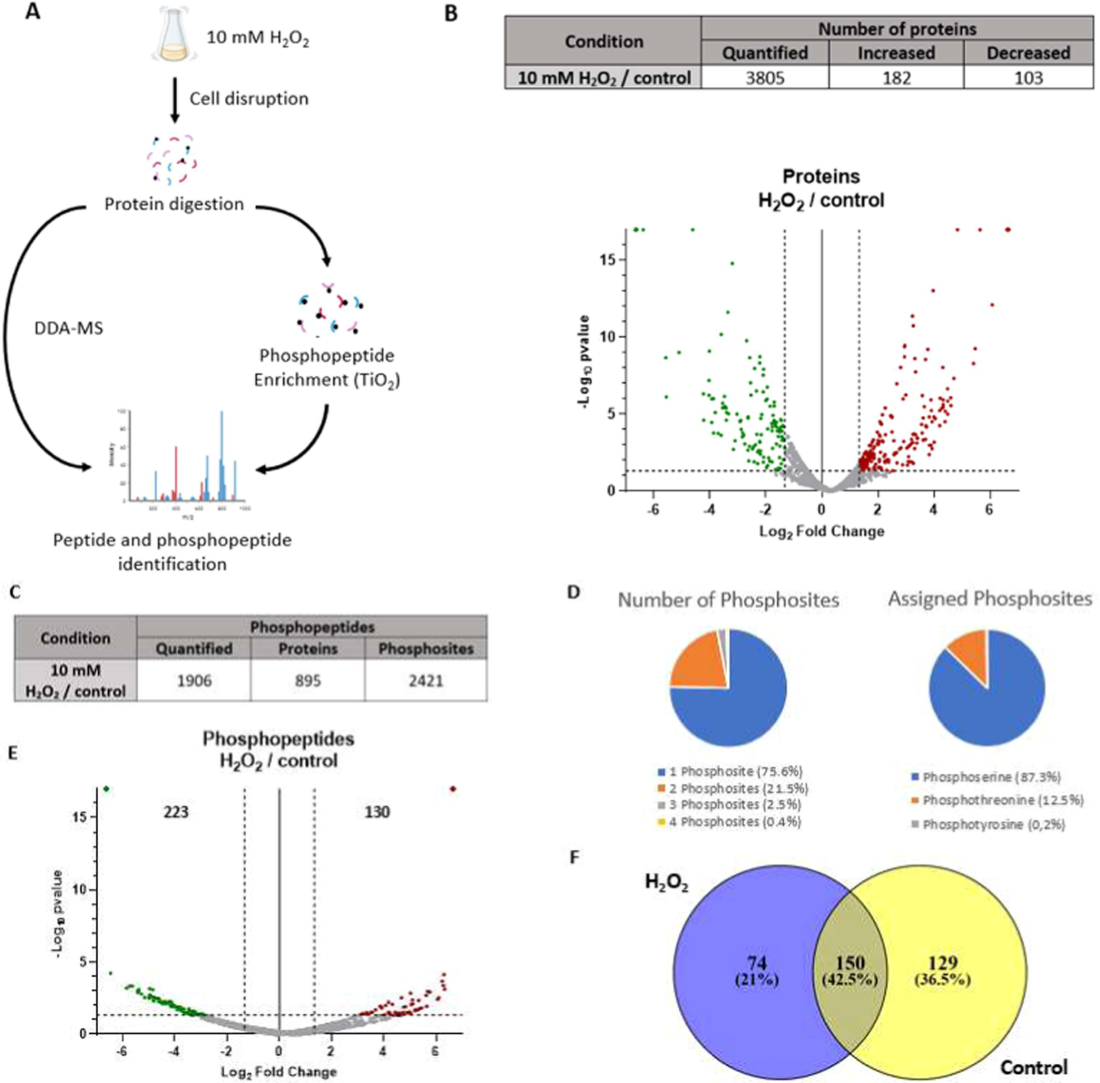
Quantitative proteomic and phosphoproteomic assay (DDA-MS) in SC5314 strain after a 200 min 10 mM H_2_O_2_treatment: (A) Workflow of quantitative proteomic and phosphoproteomic assay (DDA-MS). (B) Number of quantified proteins that changed in abundance. The volcano plot represents those proteins that significantly changed in abundance (*p*-value <0.05 and fold change >2.5) after treatment. Proteins that significantly increased or decreased in abundance are colored red and green, respectively. (C) Number of quantified phosphopeptides, number of proteins containing these phosphopeptides, and number of identified phosphosites. (D) Number of phosphosites on each phosphopeptide and percentage of total assigned phosphosites. (E) Volcano plot presenting phosphopeptides that significantly changed in abundance (*p*-value <0.05 and fold change >2.5) after the treatment. Significant changes in the phosphopeptide abundance under each condition are colored red for treated strains and green for control strains. (F) Venn diagram showing the distribution of the phosphopeptides that were only detected under treated or control condition, and those that were detected under both conditions.

The quantitative phosphoproteomic approach identified 1933 phosphopeptides, of which 1906 were quantified, corresponding to 895 proteins that contained a total of 2421 phosphosites (Figure 1C and Table S3). Around 75% of the phosphopeptides contained only 1 phosphosite, whereas 21.5, 2.5, and 0.4% presented 2, 3, and 4 phosphosites, respectively. In addition, of the total of 2421 phosphosites, 1678 (69.5%) could be assigned to a specific amino acid with a relative frequency of 87.3% for phosphoserine, 12.5% for phosphothreonine, and 0.2% for phosphotyrosine (Figure 1D). Statistical analysis using a *p*-value <0.05 and a fold change >2.5 identified 353 phosphopeptides with significant changes in abundance (Table S4). These phosphopeptides are presented in a volcano plot, highlighting those that significantly increased or decreased in abundance (Figure 1E). Phosphopeptides that were detected only under the treated or control conditions pointed out important differences in the phosphorylation pattern after the treatment (Figure 1F).

A Gene Ontology (GO) term enrichment analysis of the proteins containing phosphopeptides with significant changes in abundance (Table S4) was performed. In terms of biological processes, the phosphoproteins were enriched in the following GO terms: Biological and cellular process regulation, protein phosphorylation, chromatin remodeling, and cellular response to stimulus (Figure 2A). In terms of molecular functions, the most significant enrichment was related to nucleotide and lipid binding as well as protein kinase activity (Figure 2B). Proteins associated with each GO term are presented in Figure S1. Proteins containing phosphopeptides with significant changes in abundance were grouped into 25 clusters by using the STRING software. Notable clusters include ribosome assembly (red and dark red), transcription regulation (gold), signal transduction (green), homologous recombination (blue), DNA replication (light blue), and chaperone complex (pink). The protein kinases from the signal transduction cluster were particularly noteworthy (Figure 3).

**Figure 2:**
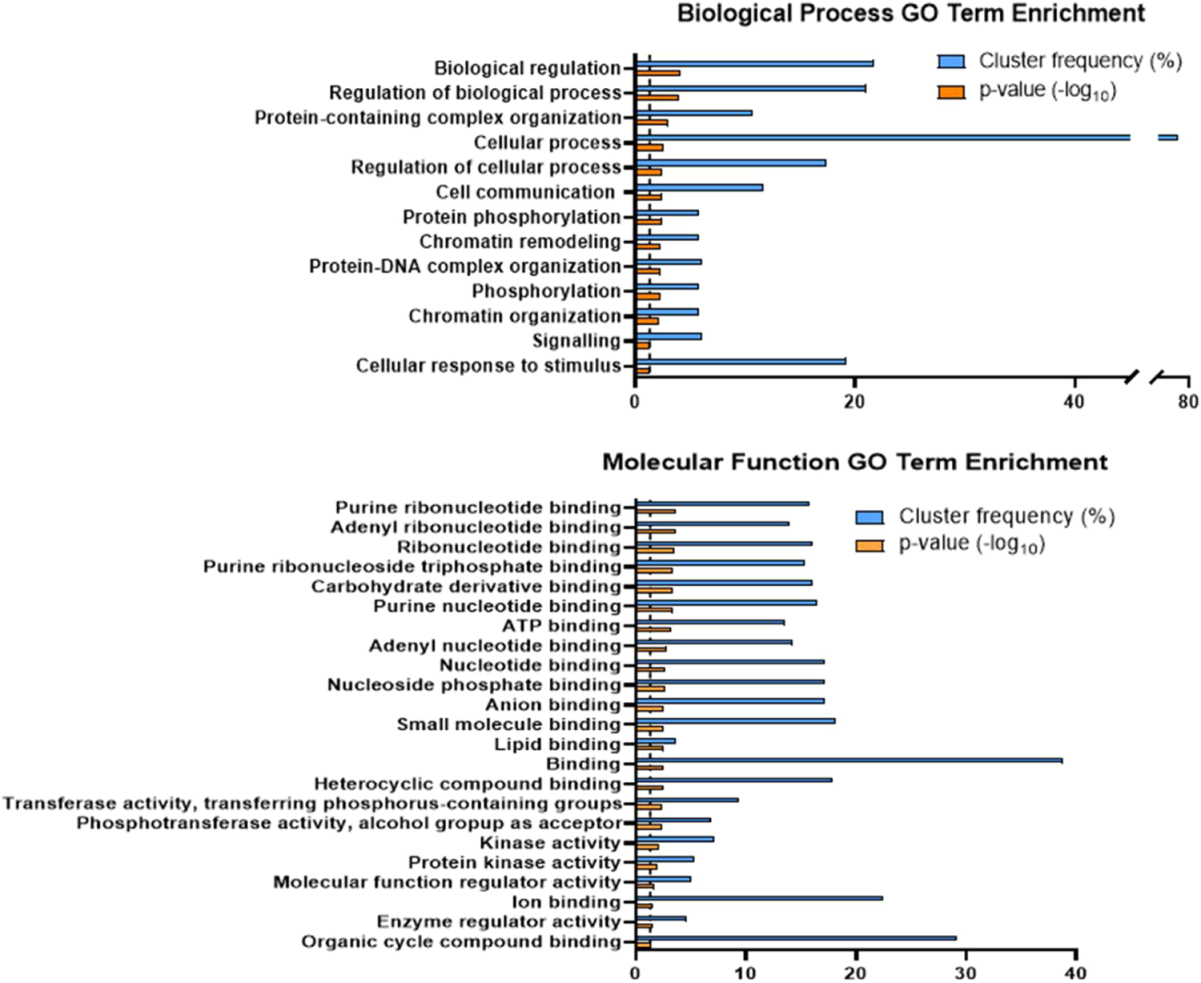
GO term enrichment of proteins containing phosphopeptides that significantly changed in abundance after 200 min of 10 mM H_2_O_2_treatment with respect to the control condition. Protein GO term enrichment is grouped by biological processes and molecular functions. Blue bars represent the cluster frequency, shown as the percentage of proteins with changes in phosphopeptide abundance with respect to the total of proteins that are associated with a specific GO term. The orange bars indicate the statistical significance, expressed as the −log_10_ *p*-value. Dotted line marks the value 1.3 corresponding to the −log_10_ *p*-value threshold for statistical significance (*p*-value = 0.05).

**Figure 3:**
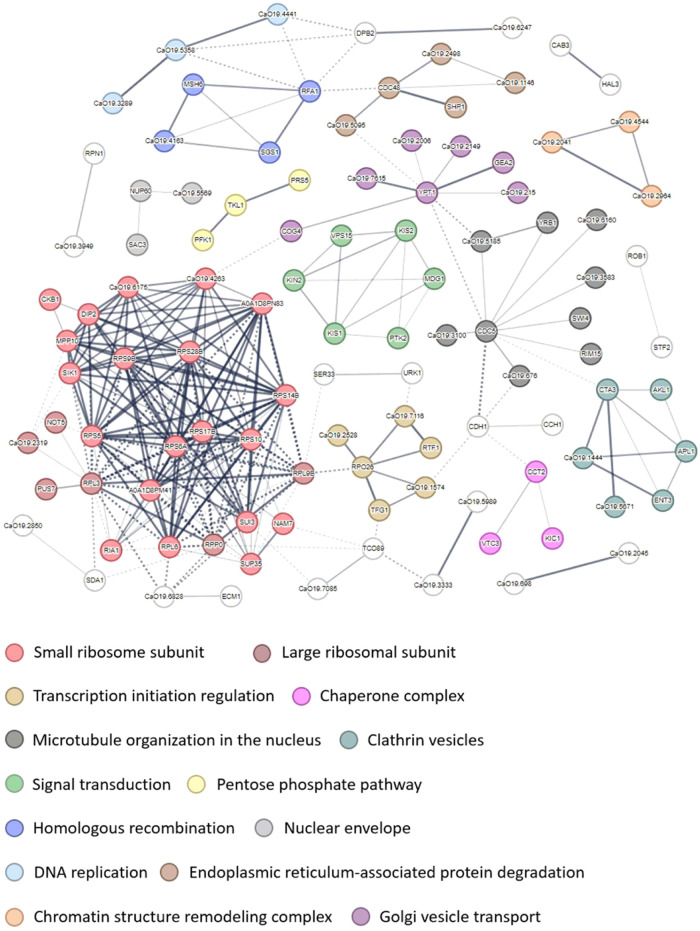
Protein clustering using STRING software for proteins in which phosphopeptides change in abundance. Line thickness indicates the strength of data support.

Proteins that exhibited significant changes in abundance overlapped with those presenting changes in their phosphopeptide abundance. This analysis identified 19 proteins that presented significant changes in both abundance and phosphorylation pattern after the 200 min treatment (Table 1). Among the 19 proteins, we found three Cap1-regulated proteins (San1, Gzf3, and orf19.7085), the metal ion reductases Fre7 and Fre10, the copper transporter Ctr1, and the damaged DNA-binding protein Rad16. Notably, while some proteins presented concordant changes between the protein abundance and phosphorylation level (measured in terms of the relative abundance of the corresponding phosphopeptides), others displayed opposing trends, as observed for Ckb1, Gzf3, Spt8, and Vps27.

**Table 1:** Proteins with a Significant Change in Both Phosphopeptides and Peptide Abundances (*p*-Value <0.05 and Fold Change >2.5) after 200 min Treatment with 10 mM H_2_O_2_, Compared to the Control Condition^a^

protein	description	phosphopeptide abundance H_2_O_2_/control	peptide abundance H_2_O_2_/control
Akr1	ankyrin-repeat protein	C	–2.53
Apl1	putative β-adaptin, large subunit of the clathrin-associated protein complex (AP-2)	C	–2.32
Cho1	putative phosphatidylserine synthase	–3.04; −3.3	3.23
Ckb1	regulatory subunit of protein kinase CK2	C	1.39
Ctr1	copper transporter; transcribed in low copper	–3.46; C	–2.1
Fre7	copper-regulated cupric reductase	–3.29; −5.41; C	–2.4
Fre10	cell-surface ferric reductase; low iron conditions	–3.31; −3.55	–1.66
Gzf3	GATA-type transcription factor; oxidative stress-induced via Cap1	C	2.05
Opt7	putative oligopeptide transporter	C; C	C
orf19.2892	unknown function; ortholog of CD36	–5.1; C	C
orf19.5799	ortholog of TPH3	–4.33; C	–1.43
orf19.7085	unknown function; oxidative stress-induced via Cap1	T	2.94
Pmc1	vacuolar calcium P-type ATPase	T	–1.63
Png2	putative peptide:N-glycanase	–4.78	–1.4
Rad16	protein that recognizes and binds damaged DNA	T	1.53
Rta2	flippase; sphingolipid long-chain base release	–3.55; −4.81; −6.46	–4.62
San1	predicted ubiquitin-protein ligase; oxidative stress-induced via Cap1	T	2.25
Spt8	subunit of the SAGA transcriptional complex	C; C	2.31
Vps27	putative ESCRT-0 complex protein with a role in multivesicular body (MVB) trafficking	C	1.49

a “T” indicates proteins or phosphopeptides only detected in treated condition; “C” indicates proteins or phosphopeptides only detected in control condition.

### Pathways Affected after 200 min of Oxidative Stress

We analyzed the pathways containing proteins with significant changes in abundance or phosphorylation pattern using the KEGG mapping software (Table S5). Pathways with the highest number of proteins as well as those potentially involved in the oxidative stress response are shown in Table 2. Ribosome and amino acid biosynthesis presented the highest numbers of proteins—involving 18 and 13 proteins, respectively—suggesting strong modulation of protein synthesis. Regarding oxidative stress signaling pathways, proteins from the pentose phosphate pathway also presented several changes in peptide and phosphopeptide abundances. We also detected numerous proteins involved in the autophagy signaling pathway, including three protein kinases (Sak1, Rim15, and Vps15; Figure S2), suggesting that autophagy is strongly modulated at this stage after the treatment. We also noted other signaling pathways linked to the oxidative stress response, such as nucleotide excision repair, mismatch repair, glutathione metabolism, and proteasome activation. Regarding the MAPK signaling pathway, we identified two distinct phosphopeptides from Bem2, one of them significantly increases in abundance, whereas the other significantly decreases in abundance.

**Table 2:** Most Relevant Signaling Pathways with Significant Protein Changes in Abundance and Phosphorylation (*p*-Value <0.05 and Fold Change >2.5) after 200 min 10 mM H_2_O_2_ Treatment

	proteins		phosphoproteins	
KEGG pathway	increased	decreased	increased	decreased
ribosome	Rps27A, Rpl37B, Rps26A, Rpl40B, Rps30		Rps17B, Rps9B, Rps10, Rps6A, orf19.1409.1, orf19.4149.1, Rpp0	Rpl6, Rps14B, Rpl3, Rpl9B, Rps5, Rps28B
biosynthesis of amino acids	Leu42, Prs4, orf19.5574	Cha1, Met15, Arg8, Arg3, Arg1	Tkl1, Prs5, Pfk1	Ser33, His7
MAPK signaling pathway	Gre2	Tus1	Bem2	Swi4, Ssk2, Bem2, Stt4
ribosome biogenesis	Rex2		Ria1, Dip2, Mpp10, Sik1	Ckb1
pentose phosphate pathway	Prs4		Pfk1, Tkl1, Prs5	Sol2
oxidative phosphorylation	Ymx6, orf19.5597.1	orf19.2343.1	Atp5	Pma1
autophagy		orf19.2733	Avt4	Sak1, Vps15, Rim15
cell cycle			Orc6, Orc2, Cdh1	Cdc5, Swi4
nucleotide excision repair		Tfb3, Tfb2	Rfa1	Rpo26
glutathione metabolism	Gst1, Glr1	Prx1		orf19.372
mismatch repair	Mlh1		Msh6, Rfa1	
proteasome	Scl1, Pre1		Rpn1	

### Cell Cycle Regulation

Cell cycle proteins presented significant changes in phosphopeptide abundance (Table 2). Phosphopeptides from Cdc5 and Swi4 decreased in abundance, whereas those from Cdh1, Orc2, and Orc6 showed an increase in abundance (Figure S3). Flow cytometry analyses were carried out to determine the cell cycle progression of SC5314 under 10 mM H_2_O_2_ treatment (Figure 4A). We observed a G2/M phase cell cycle arrest at 3 h of treatment, which partially restarted after 6 h. Growth curve analysis revealed that, while nontreated cells had nearly reached the stationary phase after 4 h, treated cells remained in the exponential phase. This finding confirms the delayed cell cycle progression caused by the G2/M phase arrest following H_2_O_2_ treatment.

**Figure 4:**
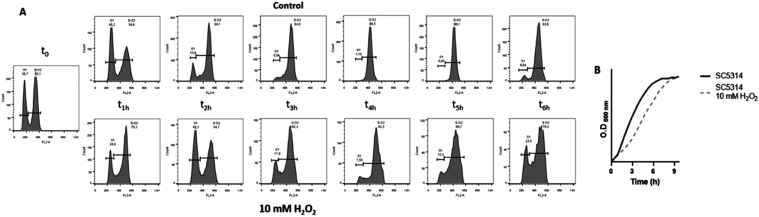
G2-M cell cycle arrest after 3–4 h 10 mM H_2_O_2_treatment: (A) Cell cycle progression of SC5314 strain under control condition and up to 6 h after 10 mM H_2_O_2_ treatment. (B) SC5314 growth curve under control condition and after 10 mM H_2_O_2_ treatment. The most representative results of three biological replicates are presented.

### MAPK Signaling

MAPK signaling pathways also presented proteins with changes in both abundance and phosphorylation patterns, indicating strong regulation of these pathways after 200 min of oxidative stress (Table 2 and Figure S4). Ssk2—the MAPKKK of the Hog1 signaling pathway—showed a decrease in phosphorylation status, consistent with the absence of Hog1 phosphorylation after 100 and 200 min H_2_O_2_ treatment, in contrast to the phosphorylation observed between 15 and 50 min (Figure 5A).

**Figure 5:**
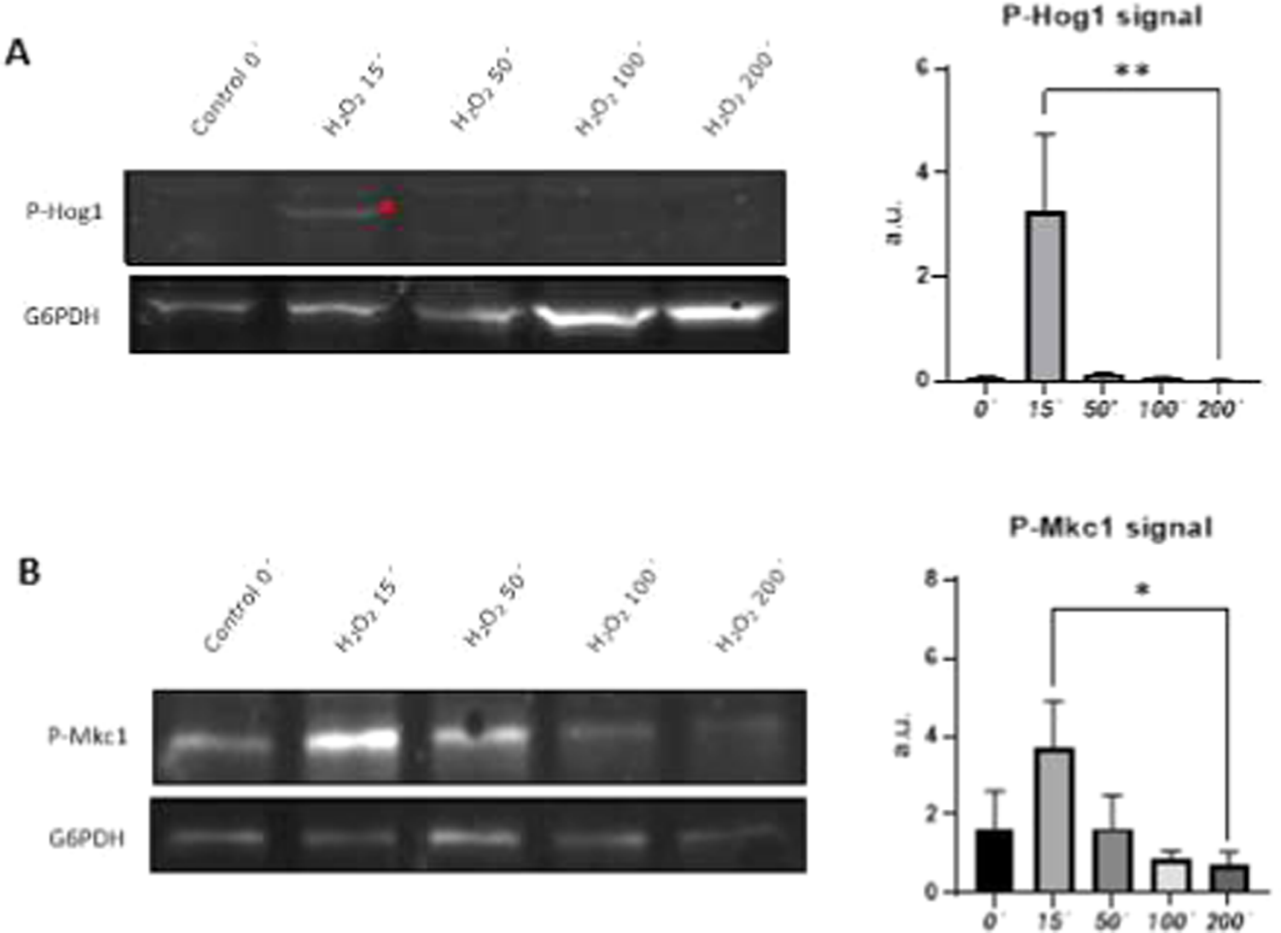
Activation status of osmotic and cell wall stress signaling pathways under treatment with 10 mM H_2_O_2_up to 200 min: (A) Western blot analysis for detection of Hog1 phosphorylation (indicated as p-Hog1) using antiphospho-p38 antibody. Glucose-6-phosphate dehydrogenase (indicated as G6PDH) was used as a loading control. Red asterisk indicates phosphorylated Hog1. Error bars indicate standard deviation. *p < 0.05, and **p < 0.01 paired *t* test. (B) Western blot analysis for detection of Mkc1 phosphorylation (indicated as p-Mkc1) using antiphospho-p42 antibody. Glucose-6-phosphate dehydrogenase (indicated as G6PDH) was used as a loading control. Error bars indicate standard deviation. *p < 0.05 and **p < 0.01 paired *t* test.

Bem2, Stt4, and Tus1 regulators of the cell wall integrity pathway also showed significant changes in phosphopeptide or protein abundances, suggesting a putative modulation of the pathway after the treatment. Mkc1MAPK phosphorylation analysis at the activation site revealed pathway activation after 15 and 50 min, recovering its basal phosphorylation status after 100 min of H_2_O_2_ treatment (Figure 5B).

### Protein Kinase and Phosphatase Interaction Network

The interaction network between the kinases and phosphatases that changed in either abundance or phosphorylation pattern is presented in Figure 6. Kin2, Kis1, Kis2, Ptk2, and Sha3 form an interaction network that putatively regulates pheromone-dependent signal transduction through Mdg1 regulation. Moreover, this network might also regulate the retrograde endosome-to-Golgi protein transport during autophagy (Vps15, Kis1, and Kis2), among other processes. Ckb1 kinase and Yvh1 phosphatase are part of the same interaction network and may be involved in the regulation of ribosomal proteins after oxidative stress. Kic1 may modulate the response to oxidative stress through Cct2 chaperonin regulation. Cdc5 is part of several kinase networks, acting alongside Akl1 in endocytosis or Rim15 in cell proliferation and autophagy. Cdc5 also forms an interaction network with orf19.4086 phosphatase, which might be involved in Ras-type GTPase Ypt1 regulation.

**Figure 6:**
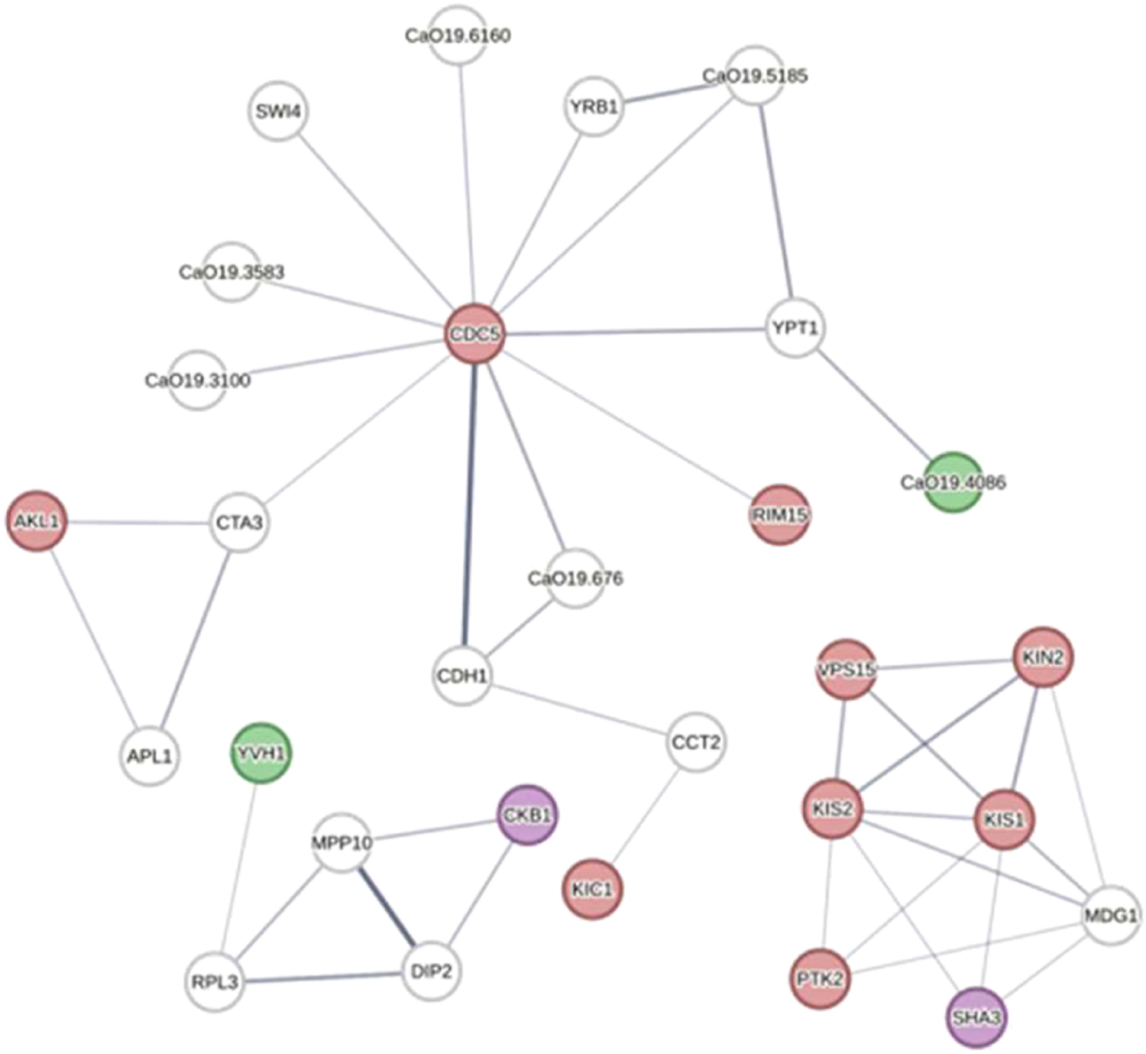
Protein kinase and phosphatase interaction network. Predicted interaction network between protein kinases and phosphatases from the proteomic assay and phosphoproteins from the phosphoproteomic assay. Protein kinases containing a phosphopeptide that significantly changed in abundance are marked in red. Protein kinases that significantly changed in abundance are marked in purple. Phosphatases that significantly changed in abundance are marked in green. Line thickness indicates the strength of data support.

Protein kinases containing phosphopeptides that significantly changed in abundance are shown in Figure 7A, with most exhibiting a significant decrease in phosphopeptide abundance. We assessed the susceptibility of kinase null mutant strains and the corresponding wild-type strain SN250 to H_2_O_2_ using a drop growth assay on YPD medium supplemented with 5 mM H_2_O_2_. The *kis1*Δ mutant strain was hypersensitive to treatment (Figure 7B). Additionally, the *npr1*Δ and *kis1*Δ strains exhibited delayed cell growth in early exponential phase liquid cultures after 10 mM H_2_O_2_ treatment (Figure 7C). However, only the *kis1*Δ mutant strain showed a statistically significant increase in the number of propidium iodide (PI)-positive dead cells due to the treatment, in concordance with the drop growth assay (Figure 7D). We also measured intracellular ROS accumulation in the mutant strains after 200 min of treatment using dihydrorhodamine 123 (DHR123). Both *kis1*Δ and *kin2*Δ strains presented increased intracellular ROS levels, although they were only statistically significant for the former (Figure 7E,F).

**Figure 7:**
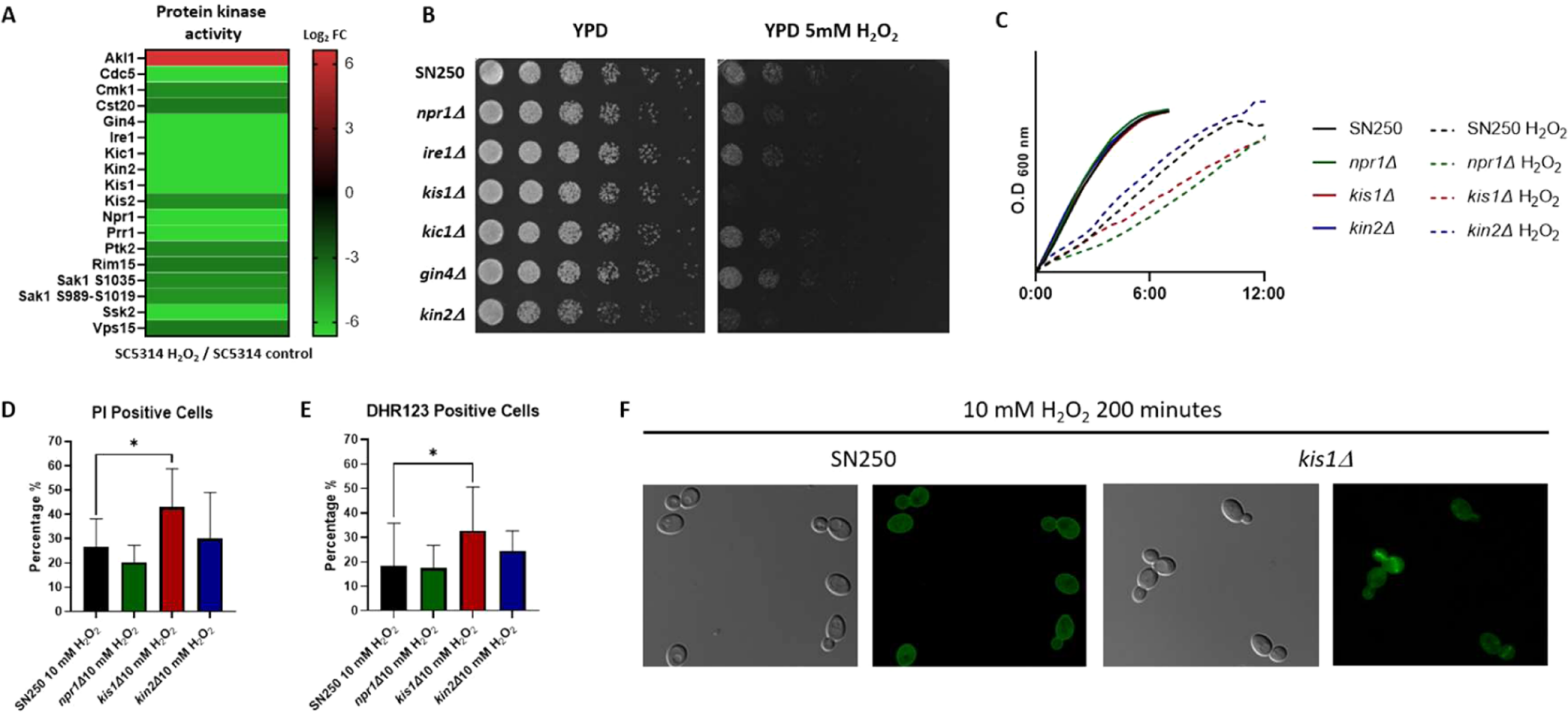
Protein kinases in the oxidative stress response: (A) Heatmap of significantly enriched protein kinase activity GO term proteins containing phosphopeptides that presented significantly changed abundance after 200 min of 10 mM H_2_O_2_ treatment. (B) Drop growth assay of SN250 wild-type strain and homozygous protein kinase mutant strains on YPD medium with 5 mM H_2_O_2_. (C) Growth curves in liquid culture of SN250, *npr1*Δ, *kis1*Δ, and *kin2*Δ strains during the early exponential phase under 10 mM H_2_O_2_. The graph shows the mean values from three biological replicates. (D) Percentage of PI-stained dead cells in SN250, *npr1*Δ, *kis1*Δ, and *kin2*Δ strains, assessed by flow cytometry after 200 min of exposure to 10 mM H_2_O_2_. (E) Percentage of intracellular ROS-positive cells in SN250, *npr1*Δ, *kis1*Δ, and *kin2*Δ strains after 200 min of 10 mM H_2_O_2_ treatment using dihydrorhodamine 123 (DHR123). (F) Fluorescence microscopy of intracellular ROS in SN250 and *kis1*Δ strains after 200 min of 10 mM H_2_O_2_ treatment using DHR123.

### Transcription Factors Involved in the Oxidative Stress Response

Further analysis using the *Candida* Genome Database of the nucleotide binding GO term-enriched proteins containing phosphopeptides that had significantly changed in abundance after the treatment identified 17 transcription factors (Figure 8A). We assessed the susceptibility of transcription factor null mutant strains from Noble’s collection to H_2_O_2_. The *gzf3*Δ mutant strain displayed slightly reduced growth in the drop assay on YPD medium supplemented with 5 mM H_2_O_2_ (Figure 8B), with delayed cell growth in liquid culture during the early exponential phase after 10 mM H_2_O_2_ treatment (Figure 8C). In addition, this strain showed a significant increase in PI-positive dead cells (Figure 8D) and a significant increase in intracellular ROS-positive cells after 200 min of 10 mM H_2_O_2_ treatment (Figure 8E,F).

**Figure 8:**
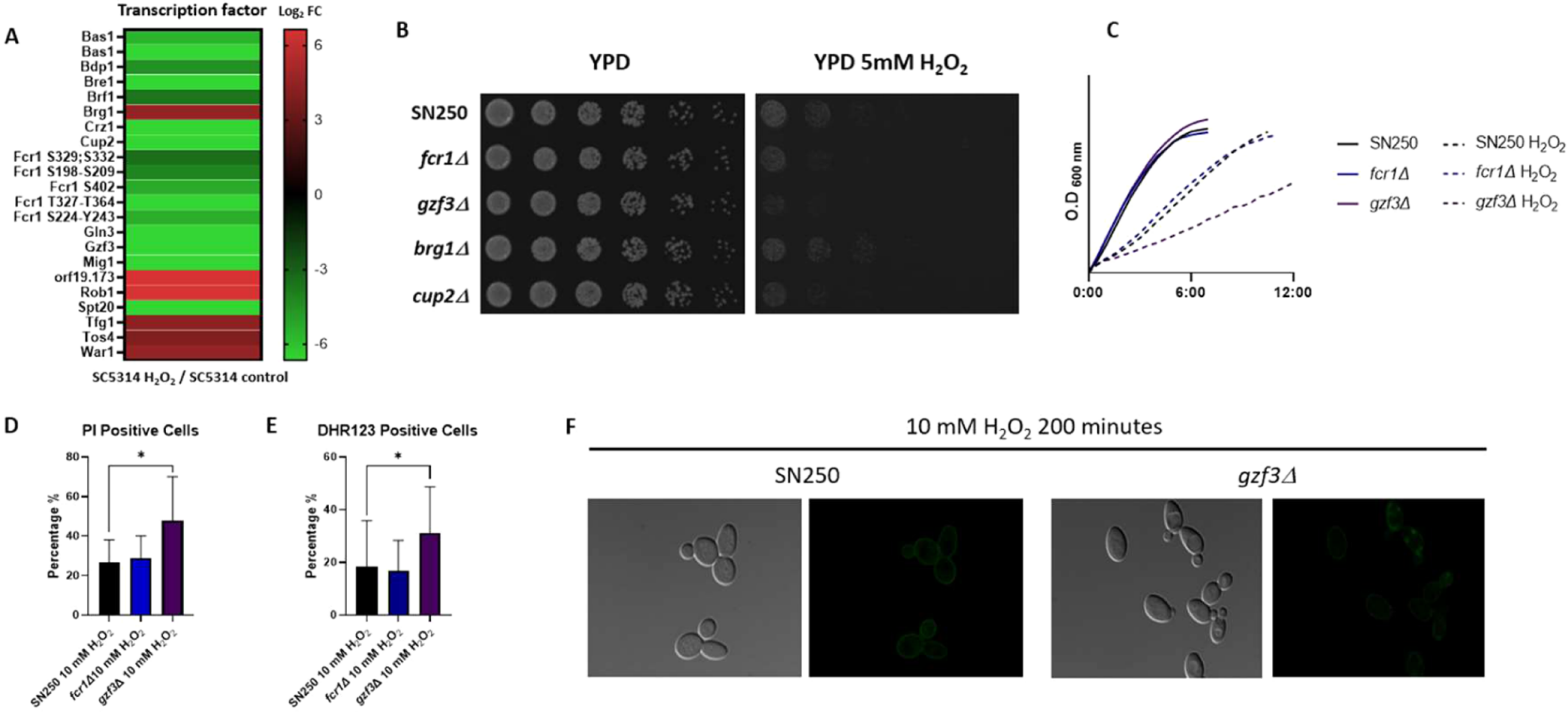
Transcription factors in the oxidative stress response: (A) Heatmap of transcription factors containing phosphopeptides that significantly changed in abundance after 200 min of 10 mM H_2_O_2_ treatment, with respect to the control condition. (B) Drop growth assay of SN250 wild-type strain and 4 homozygous transcription factor-deleted strains on YPD medium with 5 mM H_2_O_2_. (C) Growth curves in liquid culture of SN250, *fcr1*Δ, and *gzf3*Δ strains during the early exponential phase under 10 mM H_2_O_2_. The graph shows the mean values from three biological replicates. (D) Percentage of PI-positive dead cells in SN250, *fcr1*Δ, and *gzf3*Δ strains, as measured by flow cytometry after 200 min of 10 mM H_2_O_2_ treatment. (E) Percentage of intracellular ROS-positive cells in SN250, *fcr1*Δ, and *gzf3*Δ strains after 200 min of 10 mM H_2_O_2_ treatment using dihydrorhodamine 123 (DHR123). (F) Fluorescence microscopy of intracellular ROS in SN250 and *gzf3*Δ strains after 200 min of 10 mM H_2_O_2_ treatment using DHR123.

## Discussion

### Integrative Phosphoproteomic and Proteomic Analysis

Our study unveiled significant changes in protein abundance following 200 min of H_2_O_2_ treatment, consistent with previous findings.^8,9^ The novelty of our present study lies in the integration of phosphoproteomic and proteomic data through DDA-MS, thus providing a more complete picture of the cellular state after 200 min of oxidative stress. The phosphoproteomic assay quantified 1906 phosphopeptides, most of them presenting only one phosphosite, with serine being the most frequently phosphorylated amino acid (Figure 1). These results are consistent with studies on the oxidative stress responses of other pathogenic fungi.^32,33^ Phosphoprotein enrichment in nucleotide binding or chromatin remodeling GO terms (Figure 2) suggests that the significant regulation of translation via ribosomes persisted after 200 min of the treatment, together with DNA repair and proteasome functions.

Among the 19 proteins that significantly changed in both abundance and phosphorylation status (Table 1), two metal ion reductase membrane proteins (Fre7 and Fre10) and the copper transporter Ctr1 showed both reduced abundance and reduced phosphorylation. Metal ion uptake—especially iron—is necessary for the first stages of the oxidative stress response, regulating different proteins such as the transcription factor Hap43.^34,35^ Copper has a key role acting as a cofactor for superoxide dismutase Sod1.^36^ Thus, the decrease in Ctr1 abundance may reduce copper entry and, consequently, Sod1 activity, contributing to the high ROS levels observed in at 200 min after the H_2_O_2_ treatment.^5–7^ Copper is also a redox-active metal that reacts with hydrogen peroxide to create hydroxyl radicals through the Fenton-like reaction.^37^ Therefore, Ctr1 decreased both protein abundance and phosphorylation at later stages of the oxidative stress response, which might also be a protective mechanism to decrease intracellular copper levels, thus reducing additional ROS damage. Besides, metal ion reduction plays a crucial role in the oxidative stress response by facilitating the ion uptake through Ctr1.^38,39^ The diminished abundance and phosphorylation levels of Fre7 and Fre10, and the corresponding decrease in the metal ion reduction, could help to decrease the copper uptake, thus decreasing the creation of hydroxyl radicals.

### Protein Kinases in the Oxidative Stress Response

GO term enrichment in protein kinases suggests that their regulation remains involved in oxidative stress-related signaling, even after 200 min of H_2_O_2_ treatment (Figures 2A and 7A). All of the kinases (except Akl1) showed a decrease in phosphopeptide abundance. Many of these kinases are related to the oxidative stress response, such as Ssk2, Prr1, Vps15, or Rim15.^12,40–42^ *kis1*Δ—the regulatory β-subunit of the Snf1 kinase complex—showed an increased susceptibility to H_2_O_2_ (Figure 7B,D), as has been previously described.^43^ Our study also revealed previously undescribed increased intracellular ROS levels after oxidative stress, indicating that Kis1 might be implicated in ROS scavenging (Figure 7E,F).

COBALT alignment analysis (*e*-value <0.00001) of protein kinase sequences with their orthologs revealed that most of the identified phosphosites were consistent with those described in the budding yeast (Table 3). For example, Kis1 was found to contain a phosphosite in S46 that aligns with S64 in its orthologue Gal83 in . Gal83 S64 phosphorylation has been shown to be crucial for the proper functioning of the Snf1 complex in the budding yeast.^44^ Npr1—which also exhibited decreased growth in liquid medium (Figure 7C)—contains the T69 phosphosite, aligning with T31 of its ortholog, which is phosphorylated by the TORC1 kinase.^23^

**Table 3:** Protein Kinase Phosphosites in and Their Peptide Atlas Probabilities^a^

		
kinase	identified phosphosite	peptide atlas PTMProphet probability	COBALT alignment	phosphosite regulation
Akl1	**S461**	0.20–0.80	S504	DNA damage,^28^ TORC1,^21,22^ Cdk1 ^45^
Cdc5	**T25**	0.95–0.99	T23	Cdk1 regulation^26^
Cmk1	S327; S334, S335; **S336**	0.20–0.80, 0.80–0.95	Tda1 S449	DNA damage,^28,46^ osmotic stress,^27^ α factor arrest^47^
Cst20	S302; **S317**, S306, S312; **S313**	0.20–0.80, 0.80–0.95	Ste20 S192; S196	DNA damage,^46^ TORC1,^22^ Cdk1 ^45^
Gin4	**S380**	No Data	S382	Not described
Kin2	S50, S57	0.20–0.80, <0.01	No alignment	Not described
Kis1	**S46**; T47, T51	0.95–0.99, 0.20–0.80	Gal83 S64	Snf1 regulation^44^
Kis2	S198; T202	0.95–0.99	No alignment	Not described
Npr1	**T69**	0.95–0.99	T31	TORC1 ^23^
Rim15	S1183–S1190 [**S1185; S1186**]	0.20–0.99	S1047; S1048	DNA damage,^28,46^ TORC1,^22^ Cdk1 ^45^
Sak1	S989–S1019 [**S989**], **S1035**	0–0.95, 0.95–0.99	S754; S794	Not described
Ssk2	S84	0.20–0.80	No alignment	Not described
Vps15	**S963**, S965	0.95–0.99, 0.20–0.80	S952	Not described

a Specific phosphosites that align with sequence are marked in bold (COBALT alignment *e*-value < 0.00001). Brackets indicate specific phosphosites from a phosphopeptide that align with phosphosites. Phosphosite phosphorylation regulation in is indicated for each kinase.

Rim15 is a kinase involved in the oxidative stress response through the TORC1 signaling pathway in .^48^ In our study, several Rim15 phosphopeptides containing the S1185 and S1186 phosphosites showed a decrease in abundance following H_2_O_2_ treatment. These phosphosites align with S1047 and S1048 in Rim15 (Table 3), which are known to be phosphorylated by TORC1 and PKA.^22^ Under a specific stimulus, the TORC pathway is inhibited, leading to Rim15 dephosphorylation and translocation to the nucleus, regulating the expression of proteins involved in the oxidative stress response (Figure S2).^42,49,50^ Therefore, the decrease in Rim15 phosphopeptide abundance in might suggest its nuclear localization after treatment, promoting the expression of proteins related to the oxidative stress response.

Cdc5 kinase is necessary during the S-G2 phase for cell cycle progression by regulating mitosis entry and chromosome segregation.^25^ We identified a decreased abundance of the Cdc5 T25 phosphosite after the treatment. In , phosphorylation of Cdc5 at T23 by Cdk1, along with Cdh1 inhibition, is essential for Cdc5 stability, leading to cell cycle progression.^51^ The Cdc5 T25 phosphosite aligns with T23 of (Table 3), suggesting that, in , this modification modulates the cell cycle during the oxidative stress response. In accordance with this hypothesis, we observed cell cycle arrest at 3 h after 10 mM H_2_O_2_ treatment, with partial resumption at 6 h post-treatment (Figure 4). Hydrogen peroxide-mediated Hog1-delayed cell cycle has been previously described in synchronized cultures.^52^ The G2 cell cycle arrest observed following 200 min of H_2_O_2_ treatment was consistent with previously observed results in .^53^

### MAPK Phosphoproteomics

Although the Hog1 signaling pathway was turned off, we still detected increases in reductase activity proteins whose expression is regulated by this signaling pathway, such as Gre2.^54^ This indicates a strong antioxidant response, despite the inactivation of the Hog1MAPK pathway. Our identified Ssk2 S84 phosphosite does not overlap with those previously described in (Table 3); however, in , nearby phosphosites S74, S78, and T82 have been reported in response to DNA damage or Cdk1.^28,45^ Ssk2 phosphorylation at Thr1460, located on the activation loop, is crucial for its kinase activity.^55^ Nevertheless, Ssk2 S84 phosphorylation has not been previously reported as being necessary for its activation; therefore, further analyses might be performed to determine its role in Ssk2 regulation.

Hog1 activation following oxidative stress mediates cross-talk activation of the Mkc1MAPK cell wall integrity signaling pathway.^14,56^ We detected changes in the abundances of phosphopeptides from proteins involved in the regulation of Rho1 GTPase, the effector of this pathway. Bem2—a Rho1 GTPase-activating protein^57,58^ —has been previously shown to be phosphorylated by TORC1 or in response to DNA damage in .^22,28^ However, our identified Bem2 phosphosites did not align with those previously described in the budding yeast. Tus1—a GTPase-exchange factor (GEF) involved in Rho1 activation—significantly decreased in abundance after the treatment, suggesting that the cell wall integrity pathway is inactivated at this time. As shown in Figure 5B, Mkc1 was not phosphorylated after 200 min, which correlates with the decreased abundance of Tus1 and phosphorylation status of Bem2.

### Status of Transcription Factors in the Oxidative Stress Response

Among the identified transcription factors that showed a change in phosphopeptide abundance after the treatment (Figure 8A), Fcr1 and Gzf3 are regulated by Cap1.^17,18^ The results shown in Figure 8C–F suggest that Gzf3 may be involved in cell survival after oxidative stress. The phosphorylation status of oxidative stress-related transcription factors, such as Cap1, has been reported to promote gene expression.^59^ Gzf3 phosphorylation regulation might also be important for the regulation of gene expression at this point in the oxidative stress response.

COBALT alignment analysis (*e*-value <0.00001) between transcription factor phosphosites in indicated an absence of alignment with those previously described in (Table S6). In addition, most of the transcription factors identified in our study were related to filamentation (Bre1, Brg1, Bas1, Crz1, Fcr1, Gln3, Mig1, Rob1, and Tfg1) and, so do not have orthologues in . Previous studies have reported inhibition of filamentation mediated by Cek1 phosphorylation via the Hog1 kinase;^56^ however, no significant changes in phosphopeptide abundance were observed for Cek1MAPK in our study. In this manner, post-translational modifications in the described filamentation-related transcription factors could act to inhibit filamentation.

## Conclusions

At 200 min after oxidative stress, is still struggling to recover from the intense shock, as evidenced by the large number of proteins altered in terms of both abundance and phosphorylation status. The present work analyses at the same time the proteomics and the phosphoproteomics, and this can help to better understand the changes at this time point. The phosphoproteomic study has allowed the identification of new phosphosites that can be important for protein function. Our results may support future studies aimed at fully elucidating the autophagy process during the oxidative stress response, particularly through the regulation of Snf1 regulatory subunit Kis1 and Rim15 kinase. Moreover, they provide a basis for investigating the role of Cdc15 in cell cycle regulation under oxidative stress conditions as well as exploring the transcriptional function of Gzf3 in this context. This study offers valuable insights for identifying processes or proteins that could serve as potential targets for antifungal drug discovery.

## Supplementary Material





## Notes

List of 3805 quantified proteins in SC5314 strain in treated or nontreated control conditions with Proteome Discoverer software using the *Candida* Genome Database (Table S1); list of 285 proteins that significantly changed in abundance (*p*-value < 0.05 + fold change > 2.5) after 10 mM H_2_O_2_ treatment (Table S2); list of 1906 quantified phosphopeptides in SC5314 strain in treated or not treated control condition with Proteome Discoverer software using the *Candida* Genome Database (Table S3); list of 353 phosphopeptides that significantly changed in abundance (*p*-value < 0.05 + fold change > 2.5) after 10 mM H_2_O_2_ treatment (Table S4); KEGG Mapper Signaling Pathways (Table S5); and transcription factors phosphosites in and their Peptide Atlas PTMProphet probability (Table S6) (XLSX)
Heatmap of significantly enriched chromatin remodeling, cellular response to stimulus, and nucleotide binding GO term proteins (Figure S1); autophagy signaling pathway (Figure S2); cell cycle signaling pathway (Figure S3); and MAPK signaling pathway (Figure S4) (PDF)
