# Supplementary material for: Integrative Phosphoproteomic and Proteomic Analysis of Exposed to Oxidative Stress
Source: J Proteome Res. 2025 Jun 2;24(7):3484–97. doi: 10.1021/acs.jproteome.5c00137 (PMC12235712; doi:10.1021/acs.jproteome.5c00137)
Supplement: Supplementary file 2 [file pr5c00137_si_002.pdf]

Integrative phosphoproteomic and proteomic analysis of *Candida albicans* exposed to oxidative stress

Víctor Arribas,[a] Ana Borrajo,[a] María Luisa Hernáez,[b] Raquel Martínez,[a] Lucía Monteoliva,[a] Concha Gil,[a,b] Gloria Molero[a]*

[a]Department of Microbiology and Parasitology, Faculty of Pharmacy, Complutense University of Madrid (UCM), Madrid, Spain

[b]Proteomics Unit, Biological Techniques Center, Complutense University of Madrid (UCM), Madrid, Spain

* Corresponding author: Concha Gil conchagil@ucm.es

Table of contents

Figure S1 .... Page S2

Figure S2 .... Page S2

Figure S3 .... Page S3

Figure S4 .... Page S3

Supplementary Table 1 (xlsx)

Supplementary Table 2 (xlsx)

Supplementary Table 3 (xlsx)

Supplementary Table 4 (xlsx)

Supplementary Table 5 (xlsx)

Supplementary Table 6 (xlsx)

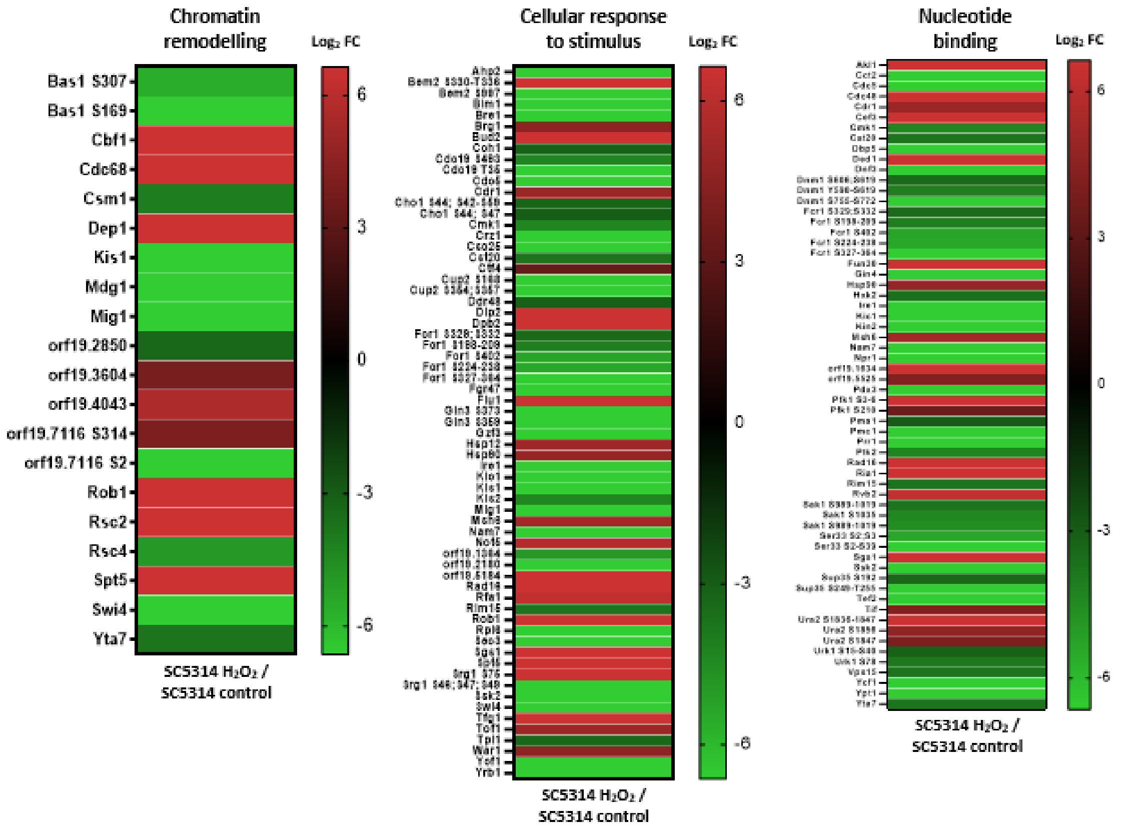

Figure S1 Heatmap of significantly enriched chromatin remodelling, cellular response to stimulus and nucleotide binding GO Term proteins.

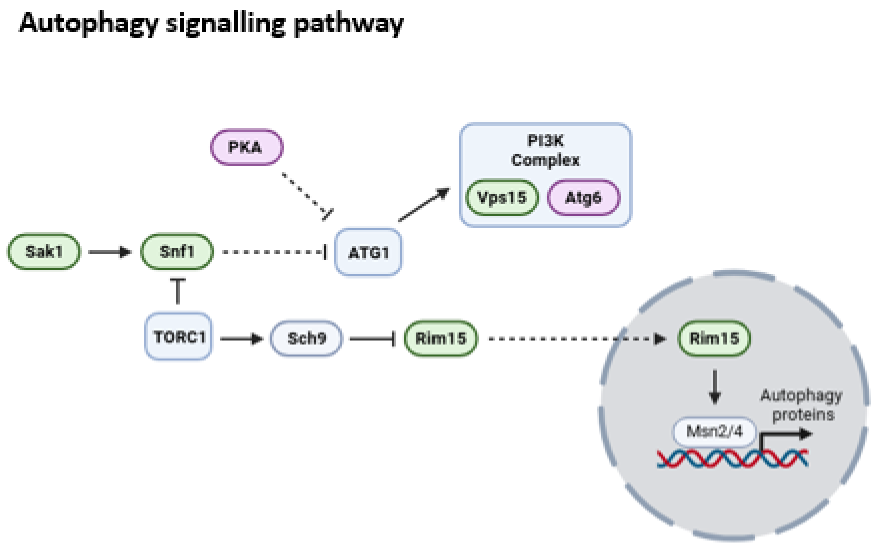

Figure S2 Autophagy signalling pathway. Proteins containing phosphopeptides with a significant decrease in abundance are marked in green. Proteins that significantly increased in abundance are marked in purple.

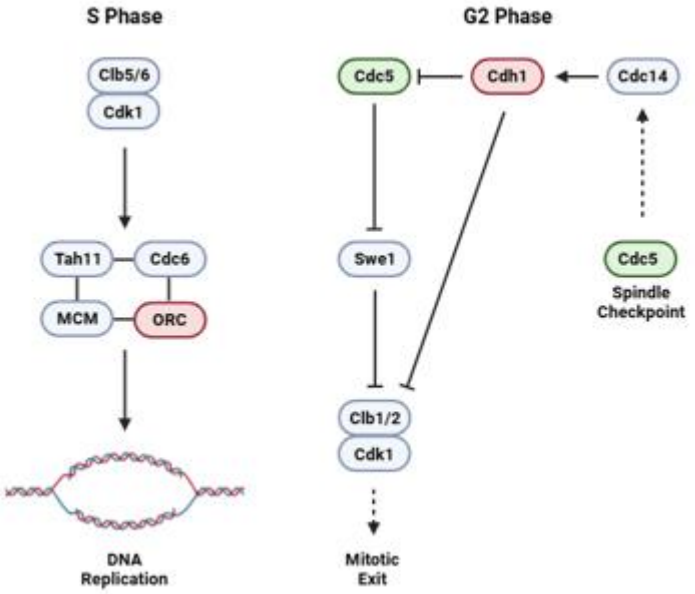

Figure S3 Cell cycle signalling pathway. Proteins containing phosphopeptides with a significant decrease in abundance are marked in green. Proteins containing phosphopeptides with a significant decrease in abundance are marked in red.

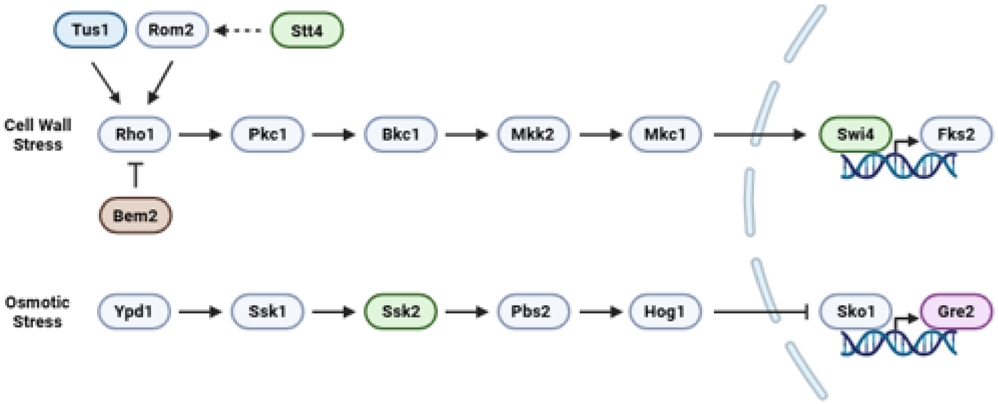

Figure S4 MAPK signalling pathway. Proteins containing phosphopeptides with a significant decrease in abundance are marked in green. Proteins containing phosphopeptides with a significant increase and decrease in abundance are marked in brown. Proteins that significantly increased in abundance are marked in purple. Proteins that significantly decreased in abundance are marked in blue.
